# Knee arthroplasty failure is associated with significant systemic multimetal exposure

**DOI:** 10.1002/ksa.70122

**Published:** 2025-10-27

**Authors:** Anna Flindt, Katrin Huesker, Georg Volk, Juliane Fuchs, André Hofer, Ulrich Schietsch, Georgi I. Wassilew, Janosch Schoon, Anastasia Rakow

**Affiliations:** ^1^ Center for Orthopaedics, Trauma Surgery and Rehabilitation Medicine University Medicine Greifswald Greifswald Germany; ^2^ Immunology Department Institute for Medical Diagnostics (IMD) Berlin Germany

**Keywords:** chromium, cobalt, multimetal quantification, revision total knee arthroplasty, total knee arthroplasty (TKA)

## Abstract

**Purpose:**

This study investigated whether patients scheduled for revision total knee arthroplasty (TKA) are systemically exposed to arthroplasty metals, and whether systemic metal levels in these patients differ depending on the implants' levels of constraint.

**Methods:**

Whole blood samples were collected from patients scheduled for revision TKA (implant group, *n* = 51) and from arthroplasty‐naïve controls (*n* = 53). Using inductively coupled plasma mass spectrometry, all TKA‐relevant metals were quantified. Differences in systemic metal levels in patients with failed unconstrained implants (*n* = 31) and constrained (*n* = 20) implants were analysed. Correlations between levels of the different arthroplasty metals were assessed, using the Mann–Whitney test, Kruskal–Wallis test with Dunn's multiple comparison test, Spearman *r* matrix, and linear regression with Spearman correlation as appropriate. A *p* < 0.05 was considered statistically significant.

**Results:**

Patients scheduled for revision TKA showed significantly higher systemic Co (*p* < 0.001), Cr (*p* < 0.001), Mo (*p* = 0.039), Ti (*p* < 0.001), Nb (*p* < 0.001) and Zr (*p* < 0.001) levels compared with controls. Failed constrained TKA implants were associated with significantly higher levels of Co (*p* = 0.002), Cr (*p* = 0.005), Ti (*p* = 0.047), Nb (*p* = 0.023) and Zr (*p* = 0.046) than detected in patients with failed unconstrained TKA implant. In patients awaiting revision of a constrained implant, whole blood levels of Co and Ti (*p* < 0.001), as well as of Zr and Ti (*p* < 0.001) significantly correlated, whereas no such correlations were observed in patients with failed unconstrained TKA implant.

**Conclusions:**

Patients with failed TKA are systemically exposed to arthroplasty metals. Correlation analyses suggest a link between the release of Co and Ti as well as of Zr and Ti in patients awaiting revision of a constrained TKA implant. Additional research is required to investigate the potential biological effects of TKA‐related metals, and to establish clinically relevant systemic threshold levels.

**Level of Evidence:**

Level II therapeutic study.

**Abbreviations**
Al: aluminium
Co: cobalt
CoCrMo: cobalt‐chromium‐molybdenum alloy
Cr: chromium
ICP‐MS: inductively coupled plasma mass spectrometry
Mo: molybdenum
MoM: metal‐on‐metal (bearing)
Nb: niobium
PMMA: polymethyl methacrylate
PS: posterior‐stabilised
Ta: tantalum
Ti: titanium
TKA: total knee arthroplasty
UC: ultra‐congruent
V: vanadium
Zr: zirconium

## INTRODUCTION

Exposure to metal particles and ions released from arthroplasty implants has been linked to different local and systemic adverse effects, primarily in context of hip arthroplasty implants with metal‐on‐metal bearings [2, 30, 33]. With the current global increase in total knee arthroplasty (TKA) procedures, concerns regarding metal release from TKA implants and related complications are growing [18, 20, 21, 32, 34, 38].

TKA and revision TKA implants are composed of multiple metals. Articulating surfaces and hinge mechanism components are predominantly made of CoCrMo alloys [12], while the bone‐side elements usually consist of titanium (Ti) alloys [30, 35]. Cones and sleeves used in revision TKA are made of tantalum, and zirconium dioxide (ZrO_2_) is widely applied in cemented TKA as it is commonly used as a radiopaque agent in polymethyl methacrylate (PMMA) bone cements [16, 28, 29].

Metal particle and ion release from TKA implants arises from various wear and corrosion processes and can be exacerbated by mechanical complications [19, 21, 27]. Local exposure to these degradation products may trigger adverse local tissue reactions, including pseudotumor formation and, much more pronounced in TKA, osteolysis and associated implant loosening, a complication of enormous clinical and socioeconomic importance [11].

Systemic exposure to arthroplasty metals has also been documented. Especially cobalt (Co) has been repeatedly associated with organ toxicity, and linked to adverse effects on the cardiovascular system, the central and peripheral nervous systems, and thyroid function [4, 5, 7, 25, 30, 31, 33, 42]. Potential immunotoxicity, cancerogenic and teratogenic effects of Co and other arthroplasty metals raise further concerns [15, 30, 40].

Despite these risks, published data on systemic metal release from TKA implants remain scarce. Existing studies are largely limited to case reports or series, focus only on Co and Cr serum levels [8, 9, 14, 30] or examine only periprosthetic tissue and/or synovial fluid [21, 26]. Consequently, there is a significant lack of knowledge regarding systemic metal exposure attributable to TKA implants, particularly in relation to the failure of such devices.

This study therefore aimed at elaborating (1) whether patients scheduled for revision TKA are systemically exposed to arthroplasty metals, (2) if whole blood levels of different arthroplasty metals correlate in these patients and (3) whether systemic metal concentrations differ according to the implants' levels of constraint.

## MATERIALS AND METHODS

### Study design, patients and outcomes

This single‐center observational study was approved by the local ethics committee (BB 178/20), and conducted in accordance with the most recent iteration of the World Medical Association Declaration of Helsinki. Between October 2020 and March 2023, consecutive eligible patients were prospectively included in this study and grouped according to implant status into either the implant group if they were scheduled for revision TKA, or into the control group if they were arthroplasty‐naïve and scheduled for primary arthroplasty. Further, the implant group was subgrouped according to the implants' extents of metallic material and metal‐on‐metal contact areas (including connecting rods). Thus, implant designs of actually different levels of constraint were summarised as follows: Cruciate‐retaining (CR) TKA, that is, the truly unconstrained implant designs, and polyethylene‐guided posterior‐stabilised (PS) and ultra‐congruent (UC) TKA, sometimes referred to as partially constrained, were pooled in the ‘unconstrained’ group. The ‘constrained’ group included both hinged and nonhinged, that is actually semiconstrained, TKA designs, in all of which at least one metal‐on‐metal contact area exists, as specified by the manufacturer. All patients aged ≥18 years and capable of consenting who were scheduled for revision or primary TKA were eligible for inclusion. Patients scheduled to undergo primary TKA due to trauma or osteonecrosis were excluded. All participants provided written informed consent. A standardised case report form was used to collect basic demographics as well as relevant medical, orthopaedic and implant data. Missing information was retrieved from digital patient records. To ensure accurate identification of implant types, implant data were obtained from clinical documentation, including so‐called implant passports. If unclear, the implant was independently assessed by three experienced arthroplasty surgeons (A.H., U.S. and G.I.W.) based on radiographs and available implantation‐related data, and a consensus was reached. This approach had to be applied in seven cases (*n* = 7) to minimise classification bias. Prerevisional anterior‐posterior radiographs of the study group's index TKA implants are shown in Figure S1.

The primary outcomes were the concentrations of aluminium (Al), Co, chromium (Cr), molybdenum (Mo), niobium (Nb), tantalum (Ta), Ti, vanadium (V) and zirconium (Zr) in whole blood of patients of the implant group and arthroplasty‐naïve controls. Secondary outcomes included correlations among these metals' levels in the implant group, and differences in systemic metal concentrations according to the implants' levels of constraint.

### Sample collection and multimetal quantification

For multimetal quantification, a single whole blood sample (≤3 mL) was collected from every participant in the course of preoperative clinical routine using standard ethylene diamine tetra acetic acid (EDTA) tubes (Vacutainer®, BD). Among the study group, the sample was taken 0–10 days preoperatively in 47 cases. As a result of short‐dated postponement of surgery, in four cases sample collection was performed 21, 55, 57 and 107 days prior to TKA revision. Whole blood samples in EDTA tubes were stored at room temperature and were analysed within 72 h of collection, as described previously [31]. In brief, samples were diluted 1:20 in high purity 0.1% NH_3_ (Suprapur, Supelco) supplemented with 0.02% Lutrol F88 (AppliChem). Subsequent multielement analyses were performed in collision/reaction cell mode by inductively coupled plasma mass spectrometry (ICP‐MS; ICapQ, Thermo Fisher) using external and internal standard calibration (Elemental Scientific). Each result represents the mean of three replicate measurements. The laboratory team was blinded to group allocation.

### Statistical analysis

Exploratory statistical analyses and data visualisation were performed using GraphPad Prism 8. Sample size was not predetermined by statistical methods since this was an exploratory study. To assess adequacy, a post hoc power analysis was conducted based on whole blood Cr levels from a previously published cohort [31]. The calculated effect size (*d* = 0.594) indicated that 48 subjects per group would provide 80% power at *α* = 0.05 (G*Power 3.1.9.7). Shapiro–Wilk test assessed the normality of the data distribution. Medians with interquartile range (IQR) were plotted since datasets were nonnormally distributed. Further information on sample size per group, error bars, and statistical tests are included in the figure legends. No samples were excluded from the analyses. Statistical significance was set at *p* < 0.05.

## RESULTS

### Patient demographics and implant data

Demographic data of the implant group and the control group were compared to identify potential confounders of systemic metal levels. There were no statistically significant differences in sex distribution between the implant and control groups (*p* = 0.2296, *χ* ^2^ test). Similarly, no significant differences were found in age, height, weight, or body mass index (BMI) between groups (Table 1). Full demographic details of all study participants are listed in Table S1.

**Table 1:** Comparison of demographic patient data (Mann–Whitney test).

	Implant group (*n* = 51)			Control group (*n* = 53)			
	Mean	Median	Range	Mean	Median	Range	*p* value
Age (years)	71.8	74.5	34.2–90.2	69.8	68.9	52.1–86.9	0.1193
Body size (cm)	171	170	153–194	170	168	148–192	0.6510
Body weight (kg)	93	90	61–149	93	87	59–167	0.6351
Body mass index (kg/m^2^)	32	30.4	22.6–49.9	32	30.8	20.5–47.8	0.9291

At sampling, the median survival of the index TKA implants was 4.7 years (range: 0.2–18.8 years). Thirty‐one patients were scheduled for revision of an unconstrained TKA, while 20 patients were awaiting revision of a constrained TKA. No statistically significant difference in implant survival was observed between these groups (median survival: unconstrained 5.5 years (0.5–18.8), constrained 4.4 years (0.2–13.6); *p* = 0.3407, Mann–Whitney test).

Regarding implant burden, 30 of 51 patients in the implant group had no additional arthroplasty implant besides the index TKA implant. Fifteen patients had one more arthroplasty implant, and five patients had two or more additional arthroplasty implants in situ. The proportion of patients with more than one arthroplasty implant in situ did not differ significantly between the constrained and unconstrained subgroups (*p* = 0.3038, *χ* ^2^ test). Details on implant types and manufacturers for all implant group participants are provided in Table S2.

### Multimetal quantification

**Figure 1:**
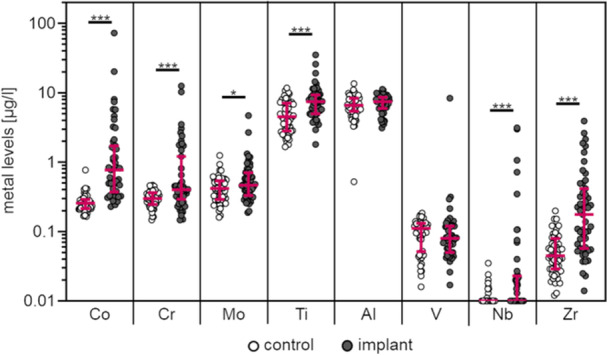
Preoperative multimetal quantification in whole blood of arthroplasty‐naïve controls (control, *n* = 53) and patients scheduled for revision total knee arthroplasty (TKA) (implant, *n* = 51). (Mann–Whitney test; median ± interquartile range; levels of significance: *p < 0.05, ***p < 0.001).

Whole blood metal levels of the implant group and the control group were compared (Figure 1, Table S3).

Patients with failed TKA showed higher levels of Co (*p* < 0.001), Cr (*p* < 0.001), Mo (*p* = 0.039), Ti (*p* < 0.001), Nb (*p* < 0.001) and Zr (*p* < 0.001) compared with controls (median metal levels [µg/L]) with (IQR): Co control, 0.26 (0.08); Co implant, 0.77 (1.34); Cr control, 0.30 (0.12); Cr implant, 0.40 (0.92); Mo control, 0.42 (0.25); Mo implant, 0.47 (0.37); Ti control, 4.52 (4.27); Ti implant, 7.45 (4.33); Nb control, 0.01 (−); Nb implant 0.01 (0.01), Zr control 0.05 (0.05); Zr implant 0.18 (0.36). An exploratory data analysis of the quantified Ta values was not performed, as the highest quantified concentration (0.08 µg/l, Table S3) was considered neglectable and most TKA implants did not contain Ta.

In addition, a subgroup comparison of whole blood metal levels across three groups was performed: arthroplasty‐naïve controls, patients with only one (index) implant, and patients with multiple arthroplasty implants in situ. No statistically significant differences in metal concentrations were observed between the subgroups (Figure S2).

In summary, systemic metal levels of patients with either one or multiple arthroplasty implants differed significantly from those in controls.

### Different levels of constraint

Patients with unconstrained and with constrained TKA implant had significantly higher levels of Co, Ti and Zr than controls. Cr and Nb levels were found to be significantly higher only in patients with failed constrained TKA implant (Table 2, Kruskal–Wallis test with Dunn's multiple comparison test).

**Table 2:** Comparison of metal levels in whole blood of arthroplasty‐naïve controls (*n* = 53) and of patients scheduled for revision of an unconstrained TKA implant (*n* = 31) or a constrained TKA (*n* = 20) (Kruskal–Wallis test with Dunn's multiple comparison test).

Analyte—group	Min (µg/l)	Median (µg/l)	Max (µg/l)	*p* value
Co—control	0.17	0.26	0.77	
Co—unconstrained	0.23	0.50	72.71	<0.001
Co—constrained	0.24	1.58	20.18	<0.001
Cr—control	0.15	0.30	0.47	
Cr—unconstrained	0.15	0.35	12.56	0.1427
Cr—constrained	0.15	1.21	10.29	<0.001
Mo—control	0.16	0.42	1.25	
Mo—unconstrained	0.19	0.46	4.69	0.2763
Mo—constrained	0.20	0.53	1.18	0.0981
Ti—control	1.66	4.52	11.74	
Ti—unconstrained	1.80	6.27	13.46	0.0080
Ti—constrained	3.76	8.13	35.10	<0.001
Nb—control	0.01	0.01	0.04	
Nb—unconstrained	0.01	0.01	0.11	0.0634
Nb—constrained	0.01	0.02	3.13	<0.001
Zr—control	0.01	0.05	0.20	
Zr—unconstrained	0.01	0.11	1.39	<0.001
Zr—constrained	0.04	0.22	3.92	<0.001

**Figure 2:**
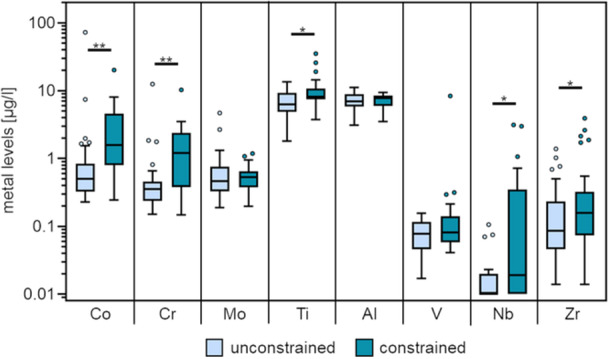
Preoperative multimetal quantification in whole blood of patients scheduled for revision of an unconstrained total knee arthroplasty (TKA) implant (*n* = 31) or a constrained TKA implant (*n* = 20). (Mann–Whitney test; Tukey box plots; levels of significance: *p < 0.05, **p < 0.01).

Based on these results, metal levels of patients with failed unconstrained TKA and failed constrained TKA were compared. These analyses revealed significantly higher levels of Co (*p* = 0.002), Cr (*p* = 0.005), Ti (*p* = 0.047), Nb (*p* = 0.023), and Zr (*p* = 0.046) in whole blood of patients with failed constrained TKA: median metal levels (µg/l) with (IQR): Co unconstrained, 0.50 (0.52); Co constrained, 1.58 (3.81); Cr unconstrained, 0.35 (0.22); Cr constrained, 1.21 (2.01); Ti unconstrained, 6.27 (4.4); Ti constrained, 8.13 (3.32); Nb unconstrained, 0.01 (0.01); Nb constrained, 0.02 (0.34); Zr unconstrained, 0.09 (0.19); Zr constrained, 0.16 (0.25) (Figure 2).

In summary, patients with failed constrained TKA implants had significantly higher systemic Co, Cr, Ti, Nb and Zr levels than those with failed unconstrained implants.

### Correlation analyses of metal levels

**Figure 3:**
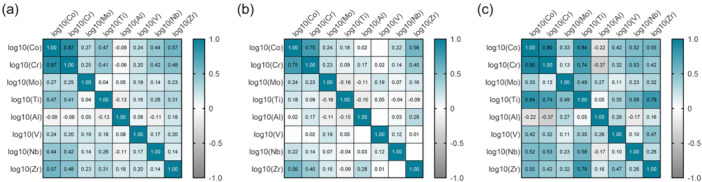
Spearman *r* matrix of multimetal correlation analyses of metal levels quantified in whole blood of patients scheduled for (a) revision total knee arthroplasty (TKA) (all patients, *n* = 51), (b) revision of an unconstrained TKA implant (*n* = 31) and (c) revision of a constrained TKA implant (*n* = 20).

To investigate how the levels of different arthroplasty metals relate to each other, a Spearman r correlation matrix was performed for each group (Figure 3). In the entire implant group, correlations of Co with Cr, Co with Ti and Co with Zr were found (Figure 3a). In the unconstrained subgroup, Co levels correlated with Cr, and Zr while correlation with Ti was weaker (Figure 3b). In the constrained subgroup, Co correlated strongly with Cr and Ti. In addition, Ti correlated with Zr (Figure 3c).

**Figure 4:**
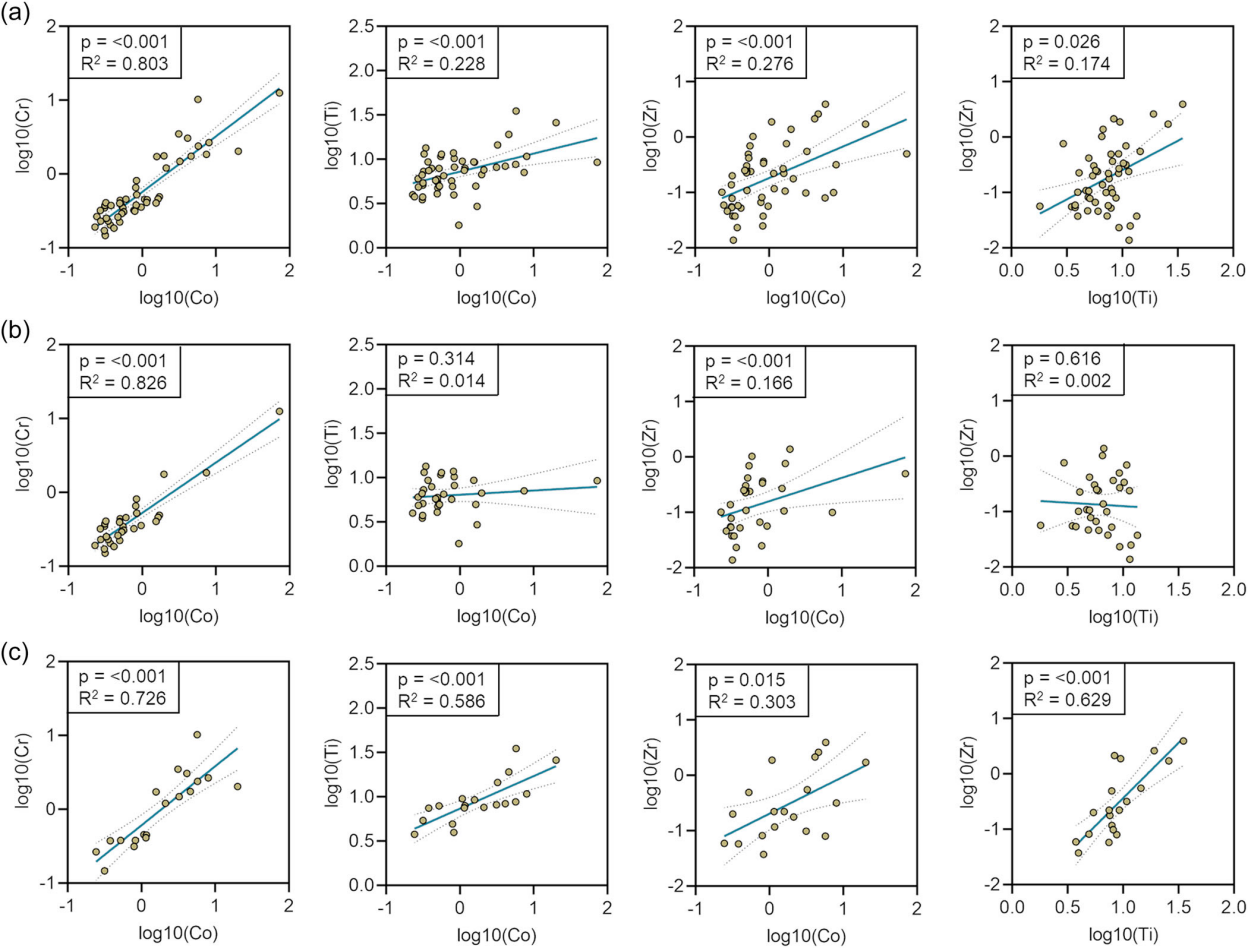
Correlation of log‐transformed metal levels quantified in whole blood of patients scheduled for (a) revision total knee arthroplasty (TKA) (all patients, *n* = 51), (b) revision of an unconstrained TKA implant (*n* = 31) and (c) revision of a constrained TKA implant (*n* = 20). (Linear regression with 95% confidence bands [dashed lines] and Spearman correlation analyses).

Based on these findings, linear regression analyses to further assess statistical significance and model fit (Figure 4a–c) were performed. Co and Cr levels significantly correlated across all subgroups. Co and Zr levels also showed significant correlations. Co and Ti did not correlate significantly in the unconstrained subgroup, but correlated in the constrained subgroup. Zr and Ti levels also significantly correlated in the constrained subgroup, but did not in the unconstrained subgroup.

Overall, Co‐Cr and Co‐Zr correlations were consistent across groups, while Co‐Ti and Zr‐Ti correlations appeared only in patients scheduled for revision of a constrained TKA.

## DISCUSSION

The most important finding of the present study is that patients with failed TKA are systemically exposed to multiple arthroplasty metals, with exposure being especially pronounced in cases of constrained TKA failure. Compared with arthroplasty‐naïve controls, patients scheduled for revision TKA had significantly higher systemic levels of Co, Cr, Mo, Ti, Nb and Zr. This was evident in patients with only the failed index TKA in situ as well as in patients with additional arthroplasty implants. Both unconstrained and constrained TKA revision patients had increased levels of Co, Ti and Zr as compared to controls. However, Cr and Nb levels were significantly elevated only in constrained TKA cases. Moreover, patients with a failed constrained TKA had significantly higher whole blood levels of Co, Cr, Ti, Nb and Zr than patients with a failed unconstrained TKA. Of note, significant correlations of Co/Cr and Co/Zr were found in both implant groups, but significant correlations of Co/Ti and Ti/Zr only in cases of constrained TKA failure.

The few studies on systemic metal exposure due to TKA implants published to date focused on quantifying the concentrations of Co, Cr, Mo and Ti [21, 22, 41]. Some looked at metal levels in patients with hinged TKA designs [12, 18]. Those analyses were mostly performed early following (revision) TKA, that is, 6 or 12 months postoperatively [22, 32], or in context of early postoperative hypersensitivity reactions possibly related to Co, Cr, Mo and Ni [17, 36]. Significant strengths of our study include that all metals relevant in revision TKA were quantified and that the quantification was conducted after chemical digestion of whole blood. In contrast to serum analyses, whole blood analyses allow the determination of the total metal content in blood, that is both cellular bound and free metals.

In TKA, articulating surfaces and hinge mechanisms are mostly made of CoCrMo alloys. Elevated Co levels have been implicated in cardiac, neurological and endocrine dysfunctions [2, 24, 42]. Due to potentially toxic effects of Co, the European Federation of National Associations of Orthopaedics and Traumatology (EFORT) and others issued a consensus statement on the management of elevated Co levels in patients with metal‐on‐metal hip implants. They recommend even asymptomatic patients with such implants and whole blood Co levels above 2–7 µg/L undergo thorough diagnostics and closer follow‐up. In cases of Co levels >20 µg/L, it is suggested to consider revision arthroplasty, even in asymptomatic patients [13]. In our cohort, 11 of 51 patients scheduled for TKA revision were found to have a Co level >2 µg/L, nine of whose constrained TKA implant had failed. A Co level >20 µg/L was diagnosed in one patient with a failed constrained TKA and in one patient with a failed unconstrained implant. However, all patients in the implant group were symptomatic. In this context, it must be emphasised that neither valid Co and other arthroplasty relevant metal thresholds for human (organ) toxicity nor evidence‐based ‘safe’ ranges of respective systemic concentrations have been defined to date. Cr, once released from the implant, is locally present as trivalent Cr which is less toxic than hexavalent Cr [43, 44]. Yet, evidence on systemic arthroprosthetic Cr exposure is inconsistent. While the toxicological profiles of Co and Cr have become increasingly better understood, the other arthroplasty metals analysed in this study have been investigated only in isolated case reports, in vitro studies or animal models [30]. Considering the potential toxicity of Ti, Al and V [1, 37, 39, 46], and this study's results, further research on possible effects in response to systemic exposure to metals used in TKA is needed. The finding that preoperatively detected systemic nickel allergy does not correlate with functional outcome after TKA [6], underscores the need to account for exposure to multiple arthroplasty metals when evaluating postoperative success.

Co/Cr and Co/Zr were positively correlated in patients with failed constrained TKA and in patients with failed unconstrained TKA, likely reflecting release from the CoCr alloys. The Co‐Zr correlation is less explicable since Zr is not part of the articulating surfaces' alloys. One hypothesis is that Co might be released first, contributing to implant loosening which causes micromovements and thus bone cement wear leading to particle release and Zr distribution. Alternatively, ZrO_2_‐containing cement particles enter the joint during implantation due to inadequate cementation or insufficient intraoperative ‘cleaning’, or later through wear, ultimately causing third body wear and thus secondary Co release.

Until now, the biocompatibility of Zr has been examined mainly in studies focusing on dental materials. Generally, ZrO₂ is considered biocompatible, and exposure to prosthetic ZrO_2_ has not been associated with acute toxicity [10]. However, it is unknown which physicochemical state of Zr is prevalent in the circulation of patients with arthroplasty implants.

Furthermore, correlations of whole blood levels of Co/Ti and of Ti/Zr, which were only evident in patients with failed constrained TKA were identified. It is well known that pairing CoCrMo with Ti alloys leads to fretting and crevice corrosion, and to the release of wear particles [45]. It remains to be investigated whether and to what extent these processes are relevant for failure of constrained TKA implants. The correlation of Co and Ti suggests that there is an increased release of Ti and Co at contact areas of the different alloys, in particular the release around hinge mechanisms may be a possible explanation. The correlation of Ti and Zr levels found only in patients with constrained TKA implants may be due to the higher total metal volume of such implants and, their usually larger cement‐metal contact surfaces. In fact, the loss of side‐to‐side and rotating motion puts additional mechanical stress on the bone‐implant interface and may thus promote implant loosening or periprosthetic fracture.

A main limitation of the performed study is that systemic metal levels were only assessed once, prohibiting assumptions about changes over time. In addition, implant and control groups were neither age‐ nor sex‐matched, and due to a relatively small overall sample size each group and subgroup had small numbers. Furthermore, the implant group was quite heterogeneous regarding index implants, limiting valid comparison across implant types. Nonetheless, considering the higher failure rates of constrained TKA as compared to unconstrained TKA [3], our results suggest future research should focus on possible interactions of Ti, Co and Zr containing degradation products and their effects on peri‐implant bone. The global increases in revision TKA, associated complications and costs [23] emphasize the importance of better understanding the causes of TKA failure.

## CONCLUSION

Systemic levels of arthroplasty metals are significantly elevated in patients with failed TKA. In particular, Co, Cr, Ti, Nb and Zr levels are significantly higher in patients awaiting revision of a constrained TKA compared with patients scheduled for revision of an unconstrained TKA. Correlation analyses suggest linked release of Co and Ti as well as of Zr and Ti in cases of constrained TKA failure. Further research is necessary to define systemic arthroplasty metal concentrations that may serve as threshold levels indicating TKA failure, and to clarify the biological and clinical consequences of all metals used in and potentially released from TKA implants.

## AUTHOR CONTRIBUTIONS

Janosch Schoon and Anastasia Rakow conceptualised and designed the study. Janosch Schoon and Anastasia Rakow wrote the study protocol. Anna Flindt, Georg Volk and Anastasia Rakow were actively involved in recruitment of study participants, and performed, supported and/or supervised clinical and implant data collection as well as sample harvesting. Katrin Huesker and Juliane Fuchs, as employees of the laboratory, performed multimetal quantification, wrote the respective paragraph under ‘methods’, and validated laboratory data. André Hofer, Ulrich Schietsch and Georgi I. Wassilew, as senior knee arthroplasty surgeons, independently verified implant identification, identified implants if respective original implant identification data were missing, and independently classified TKA implants according to the level of constraint as defined in this study. Janosch Schoon and Anna Flindt analysed the experimental data and prepared the figures. Anastasia Rakow analysed the clinical data. Anna Flindt, Janosch Schoon and Anastasia Rakow had access to all data, interpreted the results, drafted the initial manuscript and revised the manuscript. All authors critically reviewed and edited the manuscript and approved its final version.

## CONFLICT OF INTEREST STATEMENT

Katrin Huesker and Juliane Fuchs are employees at the Institute for Medical Diagnostics (IMD), Berlin, Germany. Georgi I. Wassilew serves as consultant for Mathys AG, and receives institutional funding and research support from Mathys AG and Smith & Nephew. Janosch Schoon receives institutional grants from Mathys and Smith & Nephew. Anastasia Rakow receives a research grant from the RMS Foundation outside the submitted work. The named companies did not financially support this study, had no role in study design, sample collection, data collection and analysis, decision to publish, or preparation of the manuscript. The remaining authors declare no conflict of interest.

## ETHICS STATEMENT

Following approval by the ethics committee of the University Medicine Greifswald (BB 178/20), we conducted this single‐center observational study in accordance with the most recent iteration of the World Medical Association Declaration of Helsinki between October 2020 and March 2023. All study participants provided written informed consent.

## Supporting information

Supporting information.

## Data Availability

All data generated or analysed in the course of this study are included in this published article and its supplementary information files.
