# Supplementary material for: Knee arthroplasty failure is associated with significant systemic multimetal exposure
Source: Knee Surg Sports Traumatol Arthrosc. 2025 Oct 27;34(6):2152–60. doi: 10.1002/ksa.70122 (PMC13266962; doi:10.1002/ksa.70122)
Supplement: Supplementary file 1 — Supporting information. [file KSA-34-2152-s001.docx]

**SUPPLEMENTARY INFORMATION**

**Title**

Knee arthroplasty failure is associated with significant systemic multi-metal exposure

**Table S1.** Demographic data of all study participants.

|  | age [years] | self-reported sex | body size [cm] | body weight [kg] | BMI [m^2^/kg] |
| --- | --- | --- | --- | --- | --- |
| implant group | | | | | |
| patient 1 | 79.3 | m | 163 | 75 | 28.2 |
| patient 2 | 55.7 | f | 168 | 82 | 29.1 |
| patient 3 | 68.7 | m | 175 | 116 | 37.9 |
| patient 4 | 84.7 | f | 158 | 70 | 28.0 |
| patient 5 | 47.5 | m | 194 | 103 | 27.4 |
| patient 6 | 82.8 | f | 153 | 90 | 38.4 |
| patient 7 | 68.8 | f | 168 | 90 | 31.9 |
| patient 8 | 79.6 | f | 162 | 73 | 27.8 |
| patient 9 | 89.1 | f | 165 | 89 | 32.7 |
| patient 10 | 63.9 | f | 158 | 75 | 30.0 |
| patient 11 | 71.8 | f | 168 | 70 | 24.8 |
| patient 12 | 77.2 | f | 162 | 97 | 37.0 |
| patient 13 | 51.0 | f | 160 | 67 | 26.2 |
| patient 14 | 86.9 | m | 175 | 75 | 24.5 |
| patient 15 | 83.2 | f | 155 | 82 | 34.1 |
| patient 16 | 79.6 | m | 179 | 86 | 26.8 |
| patient 17 | 62.8 | m | 186 | 105 | 30.4 |
| patient 18 | 86.5 | m | 164 | 96 | 35.7 |
| patient 19 | 74.8 | m | 170 | 103 | 35.6 |
| patient 20 | 64.9 | f | 160 | 80 | 31.3 |
| patient 21 | 43.9 | f | 176 | 70 | 22.6 |
| patient 22 | 75.6 | m | 168 | 96 | 34.0 |
| patient 23 | 80.8 | m | 166 | 111 | 40.3 |
| patient 24 | 84.4 | m | 171 | 112 | 38.3 |
| patient 25 | 90.2 | m | 180 | 75 | 23.1 |
| patient 26 | 81.7 | m | 176 | 90 | 29.1 |
| patient 27 | 55.2 | f | 167 | 89 | 31.9 |
| patient 28 | 81.0 | m | 168 | 92 | 32.6 |
| patient 29 | 34.2 | f | 164 | 61 | 22.7 |
| patient 30 | 72.9 | m | 177 | 88 | 28.1 |
| patient 31 | 66.1 | f | 155 | 120 | 49.9 |
| patient 32 | 75.3 | m | 170 | 104 | 36.0 |
| patient 33 | 64.4 | m | 178 | 95 | 30.0 |
| patient 34 | 48.4 | f | 166 | 82 | 29.8 |
| patient 35 | 72.3 | m | 174 | 103 | 34.0 |
| patient 36 | 59.5 | m | 181 | 112 | 34.2 |
| patient 37 | 86.7 | m | 192 | 92 | 25.0 |
| patient 38 | 84.3 | m | 176 | 92 | 29.7 |
| patient 39 | 73.8 | m | 182 | 86 | 26.0 |
| patient 40 | 62.7 | m | 182 | 149 | 45.0 |
| patient 41 | 83.0 | f | 156 | 115 | 47.3 |
| patient 42 | 79.0 | m | 172 | 95 | 32.1 |
| patient 43 | 72.1 | m | 186 | 100 | 28.9 |
| patient 44 | 79.5 | f | 165 | 85 | 31.2 |
| patient 45 | 60.3 | m | 178 | 144 | 45.4 |
| patient 46 | 80.5 | m | 178 | 81 | 25.6 |
| patient 47 | 69.2 | f | 170 | 87 | 30.1 |
| patient 48 | 53.6 | m | 178 | 89 | 28.1 |
| patient 49 | 83.2 | m | 168 | 80 | 28.3 |
| patient 50 | 73.6 | m | 170 | 115 | 39.8 |
| patient 51 | 74.5 | m | 184 | 110 | 32.5 |
| control group | | | | | |
| control 1 | 85.5 | m | 168 | 69 | 24.4 |
| control 2 | 70.7 | f | 162 | 87 | 33.2 |
| control 3 | 67.4 | m | 191 | 82 | 22.5 |
| control 4 | 64.1 | m | 183 | 124 | 37.0 |
| control 5 | 63.2 | f | 170 | 102 | 35.3 |
| control 6 | 78.8 | f | 168 | 60 | 21.3 |
| control 7 | 73.7 | f | 164 | 98 | 36.4 |
| control 8 | 62.3 | m | 186 | 135 | 39.0 |
| control 9 | 69.7 | f | 168 | 87 | 30.8 |
| control 10 | 83.9 | f | 157 | 59 | 23.9 |
| control 11 | 68.9 | m | 177 | 85 | 27.1 |
| control 12 | 57.7 | f | 161 | 78 | 30.1 |
| control 13 | 57.6 | f | 170 | 133 | 46.0 |
| control 14 | 78.8 | f | 160 | 91 | 35.5 |
| control 15 | 79.5 | m | 166 | 106 | 38.5 |
| control 16 | 70.6 | m | 172 | 79 | 26.7 |
| control 17 | 65.7 | m | 191 | 112 | 30.7 |
| control 18 | 64.8 | m | 180 | 105 | 32.4 |
| control 19 | 61.3 | f | 162 | 79 | 30.1 |
| control 20 | 52.1 | f | 168 | 131 | 46.4 |
| control 21 | 57.1 | m | 170 | 85 | 29.4 |
| control 22 | 63.8 | m | 186 | 140 | 40.5 |
| control 23 | 71.8 | f | 168 | 96 | 34.0 |
| control 24 | 72.4 | f | 158 | 75 | 30.0 |
| control 25 | 73.0 | m | 174 | 114 | 37.7 |
| control 26 | 80.9 | m | 168 | 77 | 27.3 |
| control 27 | 58.9 | m | 192 | 92 | 25.0 |
| control 28 | 73.7 | f | 159 | 80 | 31.6 |
| control 29 | 57.0 | m | 178 | 113 | 35.7 |
| control 30 | 65.9 | m | 187 | 167 | 47.8 |
| control 31 | 82.4 | f | 158 | 77 | 30.8 |
| control 32 | 81.7 | f | 160 | 80 | 31.3 |
| control 33 | 75.9 | f | 155 | 75 | 31.2 |
| control 34 | 67.4 | f | 158 | 69 | 27.6 |
| control 35 | 86.9 | f | 148 | 67 | 30.6 |
| control 36 | 61.5 | m | 168 | 131 | 46.4 |
| control 37 | 62.9 | m | 183 | 120 | 35.8 |
| control 38 | 76.9 | f | 157 | 64 | 26.0 |
| control 39 | 71.9 | m | 182 | 68 | 20.5 |
| control 40 | 74.6 | m | 180 | 110 | 34.0 |
| control 41 | 74.3 | f | 155 | 72 | 30.0 |
| control 42 | 64.9 | m | 174 | 91 | 30.1 |
| control 43 | 67.2 | m | 182 | 90 | 27.2 |
| control 44 | 75.2 | m | 189 | 110 | 30.8 |
| control 45 | 67.0 | m | 169 | 75 | 26.3 |
| control 46 | 63.9 | f | 166 | 72 | 26.1 |
| control 47 | 83.4 | f | 158 | 60 | 24.0 |
| control 48 | 63.3 | f | 165 | 119 | 43.7 |
| control 49 | 60.9 | m | 175 | 92 | 30.0 |
| control 50 | 57.5 | f | 174 | 87 | 28.7 |
| control 51 | 79.8 | m | 189 | 94 | 26.3 |
| control 52 | 63.9 | f | 152 | 83 | 35.9 |
| control 53 | 83.5 | f | 163 | 95 | 35.8 |

**Table S2.** Implant data.

|  | implant type | implant survival [years] | # of arthroplasty implants in situ | manufacturer | System (®) |
| --- | --- | --- | --- | --- | --- |
| patient 1 | TKA, constrained, hinged | n.a. | 1 | Implantcast | Mutars GenuX |
| patient 2 | TKA, constrained, hinged | 2.3 | 1 | LINK | Endo-Modell + Patella |
| patient 3 | TKA, semi-constrained** | 8.8 | 1 | DePuy Synthes | LCS RPS |
| patient 4 | TKA, unconstrained, PS | 5.7 | 2 | Zimmer Biomet | NexGen LPS + Patella |
| patient 5 | TKA, unconstrained, PS | 0.5 | 1 | Mathys | BalanSys PS |
| patient 6 | TKA, unconstrained, CR | n.a. | 1 | Smith & Nephew | TC Plus |
| patient 7 | TKA, unconstrained, PS | 4.7 | 1 | Zimmer Biomet | NexGen LPS |
| patient 8 | TKA, unconstrained, CR | 14.3 | 1 | ESKA Orthopaedic | Typ Genia |
| patient 9 | TKA, constrained, hinged | 13.6 | 2 | Implantcast | Mutars (distal femur) |
| patient 10 | TKA, constrained, hinged | 1.7 | 1 | LINK | Endo-Modell |
| patient 11 | TKA, constrained, hinged | 4.4 | 3 | LINK | Mega C |
| patient 12 | TKA, unconstrained, PS | 3.7 | 2 | Smith & Nephew | Journey BCS II |
| patient 13 | TKA, constrained, hinged | 2.6 | 2 | LINK | Endo-Modell SL |
| patient 14 | TKA, constrained, hinged | 0.2 | 1 | LINK | SL |
| patient 15 | TKA, constrained, hinged | 2.7 | 2 | LINK | Endo-Modell |
| patient 16 | TKA, unconstrained, PS | 8.6 | 1 | Mathys | BalanSys PS |
| patient 17 | TKA, unconstrained, UC | 1.0 | 1 | Zimmer Biomet | Persona UC |
| patient 18 | TKA, unconstrained, CR | 13.1 | 2 | Zimmer Biomet | Natural Knee II |
| patient 19 | TKA, constrained, hinged | 8.9 | 3 | LINK | Mega C |
| patient 20 | TKA, unconstrained, PS | 7.0 | 1 | DePuy Synthes | Attune |
| patient 21 | TKA, constrained, hinged | 10.2 | 1 | Implantcast | Mutars KRI |
| patient 22 | TKA, unconstrained, CR | 3.5 | 1 | Zimmer Biomet | NexGen CR |
| patient 23 | TKA, constrained, hinged | 11.1 | 2 | Implantcast | Mutars KRI |
| patient 24 | TKA, unconstrained, CR | 8.5 | 1 | Zimmer Biomet | NexGen CR |
| patient 25 | TKA, unconstrained, PS | 7.3 | 1 | Zimmer Biomet | NexGen |
| patient 26 | TKA, unconstrained, CR | 2.0 | 3 | Zimmer Biomet | NexGen |
| patient 27 | TKA, unconstrained, PS | 2.0 | 1 | Mathys | BalanSys bicondylar PS |
| patient 28 | TKA, unconstrained, UC | 12.3 | 3 | DePuy Synthes | LCS |
| patient 29 | TKA, constrained, hinged | 11.1 | 1 | n.a. | n.a. |
| patient 30 | TKA, unconstrained, CR | 14.3 | 1 | B. Braun | AESCULAP e.motion FP |
| patient 31 | TKA, unconstrained, CR | 5.1 | 2 | Zimmer Biomet | Innex CR |
| patient 32 | TKA, unconstrained, PS | 3.4 | 2 | DePuy Synthes | Attune |
| patient 33 | TKA, constrained, hinged | 11.4 | 1 | LINK | Endo-Modell Rotation |
| patient 34 | TKA, unconstrained, CR | 0.7 | 1 | DePuy Synthes | PFC |
| patient 35 | TKA, unconstrained, UC | 16.8 | 1 | DePuy Synthes | LCS |
| patient 36 | TKA, unconstrained, PS | 2.9 | 2 | Zimmer Biomet | NexGen LPS |
| patient 37 | TKA, unconstrained, PS | 11.5 | 1 | Zimmer Biomet | NexGen LPS |
| patient 38 | TKA, unconstrained, PS | 3.0 | 1 | Zimmer Biomet | NexGen LPS |
| patient 39 | TKA, unconstrained, PS | 2.6 | 1 | Zimmer Biomet | NexGen LPS |
| patient 40 | TKA, constrained, hinged | 1.7 | 2 | LINK | Mega C |
| patient 41 | TKA, constrained, hinged | 3.0 | 2 | LINK | Endo-Modell M |
| patient 42 | TKA, constrained, non-hinged | 3.9 | 3 | Zimmer Biomet | LCCK |
| patient 43 | TKA, unconstrained, UC | 9.6 | 1 | DePuy Synthes | LCS |
| patient 44 | TKA, constrained, hinged | 4.5 | 2 | LINK | Endo-Modell Rotation |
| patient 45 | TKA, unconstrained, PS | 9.5 | 2 | DePuy Synthes | Attune PS |
| patient 46 | TKA, constrained, hinged | 1.1 | 1 | Smith & Nephew | RT-Plus |
| patient 47 | TKA, unconstrained, CR | 16.4 | 2 | Smith & Nephew | TC-Plus |
| patient 48 | TKA, constrained, hinged | 4.4 | 1 | Implantcast | Mutars |
| patient 49 | TKA, unconstrained, CR | 18.8 | 3 | Ormed / DJO | Foundation |
| patient 50 | TKA, unconstrained, PS | 5.4 | 1 | Zimmer Biomet | NexGen LPS |
| patient 51 | TKA, unconstrained, CR | 2.0 | 1 | Zimmer Biomet | Persona CR |

Abbreviations and further explanations: CR, cruciate-retaining; n.a., not announced; PS, posterior-stabilized; TKA, total knee arthroplasty; UC, ultra-congruent; *including index TKA; **here classified as “constrained, non-hinged” and grouped accordingly.
Implant type, manufacturer and system each refer to the index TKA implant.


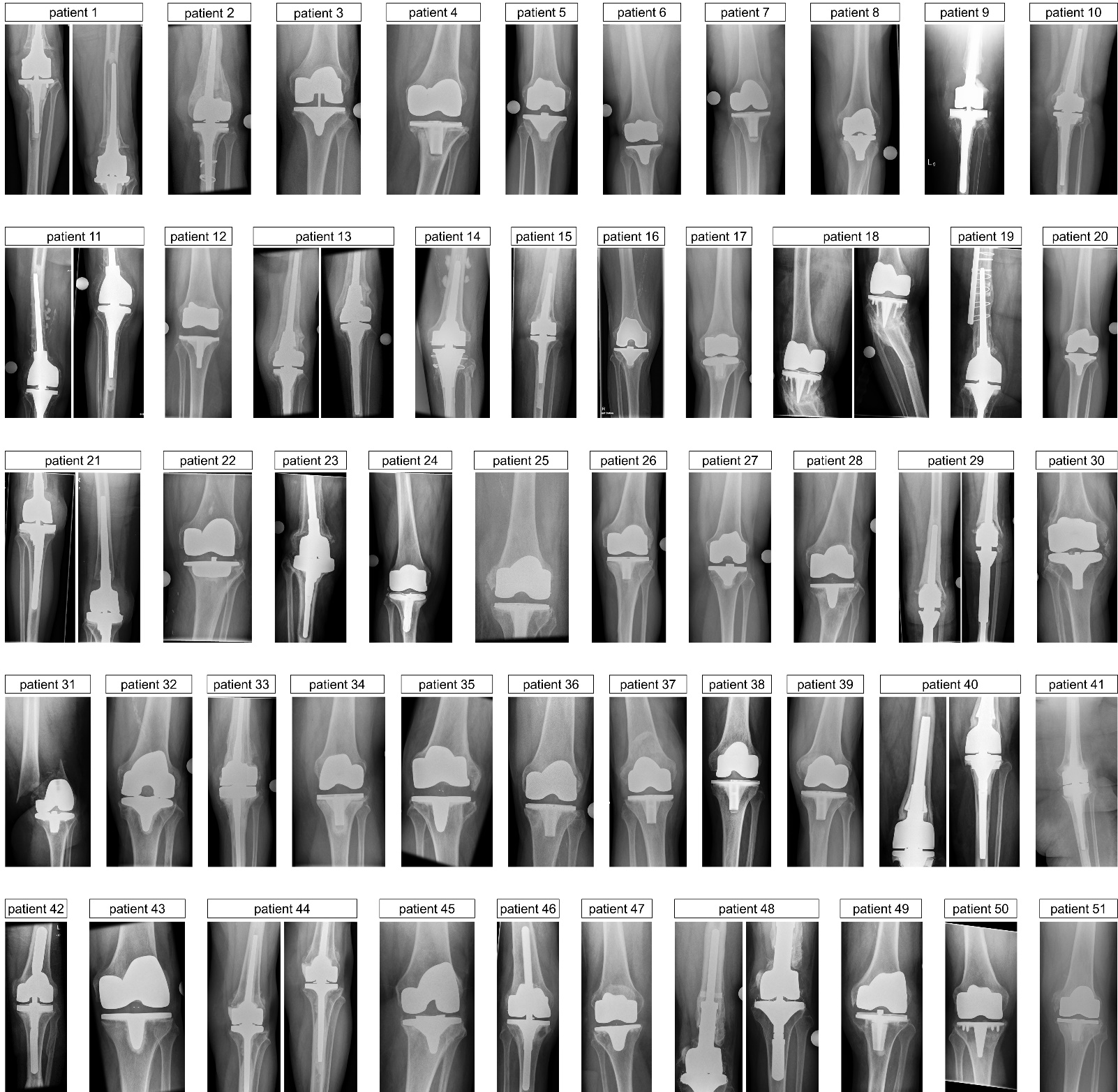


**Figure S1**. Pre-revisional anterior-posterior radiographs of the study group’s index TKA implants.

**Table S3.** Metal levels quantified by inductively coupled plasma mass spectrometry.

|  | Al [µg/l] | Cr [µg/l] | Co [µg/l] | Mo [µg/l] | Nb [µg/l] | Ta [µg/l] | Ti [µg/l] | V [µg/l] | Zr [µg/l] |
| --- | --- | --- | --- | --- | --- | --- | --- | --- | --- |
| implant group | | | | | | | | | |
| patient 1 | 3.63 | 1.74 | 4.61 | 0.71 | 0.04 | 0.03 | 19.09 | 0.21 | 2.62 |
| patient 2 | 6.16 | 3.07 | 4.12 | 0.45 | 0.01 | 0.01 | 8.35 | 8.35 | 2.14 |
| patient 3 | 5.77 | 0.19 | 0.23 | 0.47 | 0.01 | 0.01 | 3.96 | 0.02 | 0.10 |
| patient 4 | 6.61 | 1.77 | 1.98 | 0.58 | 0.02 | 0.03 | 6.70 | 0.14 | 1.39 |
| patient 5 | 3.11 | 0.20 | 0.37 | 0.39 | 0.02 | 0.02 | 9.31 | 0.12 | 0.02 |
| patient 6 | 7.71 | 0.22 | 0.50 | 0.80 | 0.01 | 0.01 | 5.52 | 0.13 | 0.30 |
| patient 7 | 6.42 | 0.32 | 0.77 | 0.50 | 0.01 | 0.01 | 5.70 | 0.08 | 0.07 |
| patient 8 | 5.06 | 12.56 | 72.71 | 2.68 | 0.02 | 0.01 | 9.22 | 0.12 | 0.50 |
| patient 9 | 8.18 | 2.03 | 20.18 | 0.95 | 1.07 | 0.01 | 25.91 | 0.32 | 1.71 |
| patient 10 | 4.11 | 2.66 | 8.05 | 0.60 | 2.99 | 0.04 | 10.77 | 0.06 | 0.32 |
| patient 11 | 9.45 | 0.45 | 1.08 | 0.53 | 3.13 | 0.01 | 9.50 | 0.12 | 1.88 |
| patient 12 | 6.02 | 0.36 | 0.97 | 0.45 | 0.02 | 0.02 | 1.80 | 0.05 | 0.06 |
| patient 13 | 7.65 | 0.38 | 0.52 | 0.43 | 0.02 | 0.08 | 7.89 | 0.07 | 0.49 |
| patient 14 | 8.36 | 0.38 | 0.83 | 0.62 | 0.01 | 0.01 | 3.95 | 0.14 | 0.04 |
| patient 15 | 7.99 | 0.45 | 1.17 | 0.48 | 0.11 | 0.03 | 7.92 | 0.09 | 0.12 |
| patient 16 | 9.01 | 0.44 | 0.50 | 0.87 | 0.11 | 0.05 | 4.71 | 0.11 | 0.11 |
| patient 17 | 10.90 | 0.35 | 0.55 | 0.29 | 0.01 | 0.01 | 10.79 | 0.07 | 0.69 |
| patient 18 | 6.25 | 0.19 | 0.42 | 4.69 | 0.07 | 0.01 | 7.88 | 0.16 | 0.05 |
| patient 19 | 9.39 | 2.39 | 5.78 | 0.65 | 0.02 | 0.01 | 35.10 | 0.29 | 3.92 |
| patient 20 | 11.22 | 0.45 | 0.60 | 0.78 | 0.02 | 0.02 | 6.51 | 0.08 | 1.03 |
| patient 21 | 5.93 | 10.29 | 5.71 | 0.31 | 0.35 | 0.04 | 8.77 | 0.08 | 0.08 |
| patient 22 | 9.83 | 0.31 | 0.57 | 0.70 | 0.01 | 0.01 | 11.41 | 0.04 | 0.24 |
| patient 23 | 7.77 | 1.72 | 1.58 | 1.08 | 0.72 | 0.01 | 9.22 | 0.05 | 0.22 |
| patient 24 | 9.33 | 0.35 | 0.31 | 0.97 | 0.01 | 0.02 | 3.48 | 0.04 | 0.06 |
| patient 25 | 7.86 | 0.38 | 0.31 | 0.39 | 0.01 | 0.01 | 3.75 | 0.09 | 0.05 |
| patient 26 | 7.29 | 0.40 | 0.50 | 0.47 | 0.01 | 0.01 | 5.96 | 0.07 | 0.24 |
| patient 27 | 9.73 | 0.41 | 0.32 | 0.40 | 0.01 | 0.01 | 7.30 | 0.12 | 0.04 |
| patient 28 | 6.89 | 0.49 | 1.72 | 0.47 | 0.01 | 0.01 | 2.93 | 0.05 | 0.76 |
| patient 29 | 6.96 | 0.26 | 0.24 | 0.58 | 0.01 | 0.01 | 3.75 | 0.04 | 0.06 |
| patient 30 | 6.29 | 0.40 | 0.49 | 1.32 | 0.01 | 0.01 | 4.07 | 0.14 | 0.23 |
| patient 31 | 6.78 | 0.52 | 0.84 | 0.41 | 0.07 | 0.03 | 8.14 | 0.04 | 0.36 |
| patient 32 | 8.64 | 0.29 | 0.53 | 0.27 | 0.01 | 0.02 | 4.98 | 0.13 | 0.42 |
| patient 33 | 8.50 | 0.31 | 0.79 | 0.57 | 0.01 | 0.01 | 4.93 | 0.12 | 0.08 |
| patient 34 | 5.09 | 0.32 | 0.27 | 0.47 | 0.01 | 0.01 | 6.03 | 0.12 | 0.05 |
| patient 35 | 7.66 | 0.82 | 0.84 | 0.25 | 0.01 | 0.01 | 10.22 | 0.09 | 0.34 |
| patient 36 | 3.91 | 0.23 | 0.35 | 0.46 | 0.01 | 0.01 | 13.45 | 0.07 | 0.04 |
| patient 37 | 9.53 | 0.46 | 1.65 | 0.41 | 0.02 | 0.02 | 4.98 | 0.05 | 0.11 |
| patient 38 | 7.00 | 0.65 | 0.82 | 0.31 | 0.02 | 0.02 | 11.76 | 0.07 | 0.02 |
| patient 39 | 6.91 | 0.15 | 0.32 | 0.32 | 0.02 | 0.01 | 5.10 | 0.10 | 0.08 |
| patient 40 | 3.53 | 1.21 | 2.12 | 0.37 | 0.02 | 0.06 | 7.61 | 0.06 | 0.18 |
| patient 41 | 8.88 | 1.49 | 3.23 | 1.18 | 0.01 | 0.01 | 14.48 | 0.06 | 0.55 |
| patient 42 | 7.69 | 0.37 | 0.38 | 0.47 | 0.01 | 0.01 | 7.45 | 0.05 | 0.06 |
| patient 43 | 7.44 | 0.23 | 0.28 | 0.33 | 0.02 | 0.06 | 4.86 | 0.04 | 0.05 |
| patient 44 | 8.68 | 0.15 | 0.32 | 0.29 | 0.01 | 0.03 | 5.40 | 0.07 | 0.20 |
| patient 45 | 9.91 | 0.17 | 0.31 | 0.19 | 0.01 | 0.01 | 6.63 | 0.08 | 0.14 |
| patient 46 | 7.97 | 0.41 | 1.15 | 0.20 | 0.01 | 0.01 | 7.48 | 0.08 | 0.22 |
| patient 47 | 8.58 | 1.85 | 7.42 | 0.71 | 0.01 | 0.01 | 7.12 | 0.08 | 0.10 |
| patient 48 | 3.82 | 3.50 | 3.13 | 0.32 | 0.03 | 0.04 | 8.13 | 0.08 | 0.10 |
| patient 49 | 3.80 | 0.40 | 1.55 | 1.22 | 0.01 | 0.01 | 9.31 | 0.05 | 0.27 |
| patient 50 | 4.13 | 0.25 | 0.33 | 0.27 | 0.01 | 0.01 | 11.49 | 0.05 | 0.01 |
| patient 51 | 4.99 | 0.26 | 0.47 | 0.26 | 0.01 | 0.01 | 5.76 | 0.03 | 0.25 |
| control group | | | | | | | | | |
| control 1 | 5.44 | 0.34 | 0.26 | 0.28 | 0.01 | 0.01 | 10.78 | 0.14 | 0.02 |
| control 2 | 9.84 | 0.24 | 0.24 | 0.52 | 0.01 | 0.01 | 3.13 | 0.04 | 0.09 |
| control 3 | 4.63 | 0.24 | 0.21 | 0.25 | 0.01 | 0.01 | 2.12 | 0.05 | 0.01 |
| control 4 | 4.59 | 0.27 | 0.26 | 0.30 | 0.01 | 0.01 | 2.54 | 0.12 | 0.02 |
| control 5 | 3.98 | 0.33 | 0.39 | 0.93 | 0.01 | 0.01 | 1.90 | 0.04 | 0.07 |
| control 6 | 7.95 | 0.16 | 0.28 | 0.48 | 0.02 | 0.01 | 2.40 | 0.11 | 0.03 |
| control 7 | 6.88 | 0.15 | 0.21 | 0.29 | 0.02 | 0.02 | 1.80 | 0.03 | 0.07 |
| control 8 | 4.52 | 0.23 | 0.21 | 0.51 | 0.01 | 0.01 | 1.66 | 0.11 | 0.08 |
| control 9 | 5.74 | 0.16 | 0.27 | 0.52 | 0.03 | 0.03 | 2.12 | 0.04 | 0.12 |
| control 10 | 6.49 | 0.21 | 0.21 | 0.33 | 0.01 | 0.01 | 3.22 | 0.03 | 0.01 |
| control 11 | 9.26 | 0.32 | 0.30 | 0.42 | 0.02 | 0.01 | 2.51 | 0.04 | 0.04 |
| control 12 | 4.10 | 0.33 | 0.41 | 0.34 | 0.02 | 0.01 | 3.61 | 0.05 | 0.09 |
| control 13 | 3.35 | 0.24 | 0.18 | 0.26 | 0.01 | 0.01 | 2.24 | 0.02 | 0.06 |
| control 14 | 7.58 | 0.21 | 0.27 | 0.39 | 0.01 | 0.01 | 2.76 | 0.02 | 0.06 |
| control 15 | 8.95 | 0.19 | 0.36 | 0.25 | 0.01 | 0.01 | 2.74 | 0.05 | 0.06 |
| control 16 | 5.89 | 0.29 | 0.22 | 0.55 | 0.01 | 0.01 | 7.58 | 0.14 | 0.04 |
| control 17 | 9.37 | 0.25 | 0.41 | 0.24 | 0.01 | 0.01 | 9.67 | 0.09 | 0.04 |
| control 18 | 6.66 | 0.22 | 0.19 | 0.17 | 0.01 | 0.01 | 7.98 | 0.12 | 0.04 |
| control 19 | 5.44 | 0.32 | 0.21 | 0.28 | 0.01 | 0.01 | 10.08 | 0.14 | 0.02 |
| control 20 | 3.28 | 0.30 | 0.41 | 0.42 | 0.01 | 0.01 | 9.13 | 0.14 | 0.05 |
| control 21 | 0.52 | 0.39 | 0.31 | 0.28 | 0.01 | 0.01 | 11.74 | 0.11 | 0.02 |
| control 22 | 13.56 | 0.39 | 0.23 | 0.50 | 0.01 | 0.01 | 8.28 | 0.13 | 0.02 |
| control 23 | 9.84 | 0.41 | 0.22 | 0.47 | 0.01 | 0.01 | 7.17 | 0.17 | 0.03 |
| control 24 | 4.13 | 0.36 | 0.23 | 0.58 | 0.01 | 0.01 | 7.30 | 0.11 | 0.03 |
| control 25 | 7.94 | 0.43 | 0.24 | 0.76 | 0.01 | 0.01 | 6.78 | 0.17 | 0.06 |
| control 26 | 3.84 | 0.35 | 0.23 | 0.38 | 0.01 | 0.01 | 7.32 | 0.11 | 0.03 |
| control 27 | 5.33 | 0.28 | 0.28 | 0.32 | 0.01 | 0.01 | 4.96 | 0.08 | 0.02 |
| control 28 | 6.34 | 0.47 | 0.35 | 0.55 | 0.01 | 0.01 | 3.68 | 0.11 | 0.04 |
| control 29 | 5.99 | 0.39 | 0.26 | 0.64 | 0.01 | 0.01 | 4.76 | 0.12 | 0.03 |
| control 30 | 5.53 | 0.29 | 0.26 | 1.25 | 0.01 | 0.01 | 4.91 | 0.09 | 0.04 |
| control 31 | 5.38 | 0.23 | 0.26 | 0.57 | 0.01 | 0.02 | 4.47 | 0.05 | 0.06 |
| control 32 | 5.71 | 0.22 | 0.21 | 0.29 | 0.01 | 0.01 | 2.90 | 0.03 | 0.04 |
| control 33 | 10.29 | 0.28 | 0.77 | 0.42 | 0.01 | 0.02 | 6.02 | 0.11 | 0.04 |
| control 34 | 8.55 | 0.26 | 0.27 | 0.37 | 0.01 | 0.01 | 7.02 | 0.11 | 0.06 |
| control 35 | 8.39 | 0.29 | 0.26 | 0.35 | 0.01 | 0.01 | 7.27 | 0.12 | 0.09 |
| control 36 | 9.79 | 0.29 | 0.26 | 0.20 | 0.01 | 0.01 | 6.81 | 0.10 | 0.05 |
| control 37 | 8.35 | 0.37 | 0.17 | 0.21 | 0.01 | 0.01 | 8.36 | 0.09 | 0.08 |
| control 38 | 6.61 | 0.29 | 0.22 | 0.33 | 0.01 | 0.02 | 6.61 | 0.12 | 0.05 |
| control 39 | 7.49 | 0.30 | 0.22 | 0.56 | 0.01 | 0.01 | 4.52 | 0.19 | 0.03 |
| control 40 | 6.83 | 0.35 | 0.19 | 0.26 | 0.01 | 0.01 | 4.08 | 0.17 | 0.02 |
| control 41 | 6.55 | 0.32 | 0.28 | 0.53 | 0.01 | 0.01 | 4.77 | 0.16 | 0.04 |
| control 42 | 7.05 | 0.29 | 0.26 | 0.16 | 0.01 | 0.01 | 4.67 | 0.16 | 0.02 |
| control 43 | 7.76 | 0.38 | 0.22 | 0.38 | 0.01 | 0.01 | 4.97 | 0.13 | 0.03 |
| control 44 | 5.87 | 0.27 | 0.21 | 0.51 | 0.01 | 0.01 | 5.14 | 0.13 | 0.06 |
| control 45 | 4.67 | 0.33 | 0.17 | 0.59 | 0.01 | 0.01 | 5.89 | 0.14 | 0.08 |
| control 46 | 9.18 | 0.36 | 0.42 | 0.43 | 0.01 | 0.01 | 3.31 | 0.08 | 0.11 |
| control 47 | 9.21 | 0.45 | 0.33 | 0.53 | 0.01 | 0.01 | 2.84 | 0.12 | 0.12 |
| control 48 | 11.08 | 0.36 | 0.22 | 0.59 | 0.01 | 0.01 | 4.19 | 0.12 | 0.15 |
| control 49 | 7.63 | 0.35 | 0.17 | 0.33 | 0.01 | 0.01 | 3.91 | 0.10 | 0.15 |
| control 50 | 8.06 | 0.40 | 0.25 | 0.76 | 0.01 | 0.01 | 2.80 | 0.13 | 0.10 |
| control 51 | 5.77 | 0.46 | 0.24 | 0.49 | 0.01 | 0.01 | 2.51 | 0.08 | 0.12 |
| control 52 | 6.16 | 0.39 | 0.32 | 0.32 | 0.01 | 0.01 | 3.82 | 0.03 | 0.20 |
| control 53 | 6.67 | 0.25 | 0.30 | 0.62 | 0.02 | 0.02 | 3.57 | 0.13 | 0.03 |


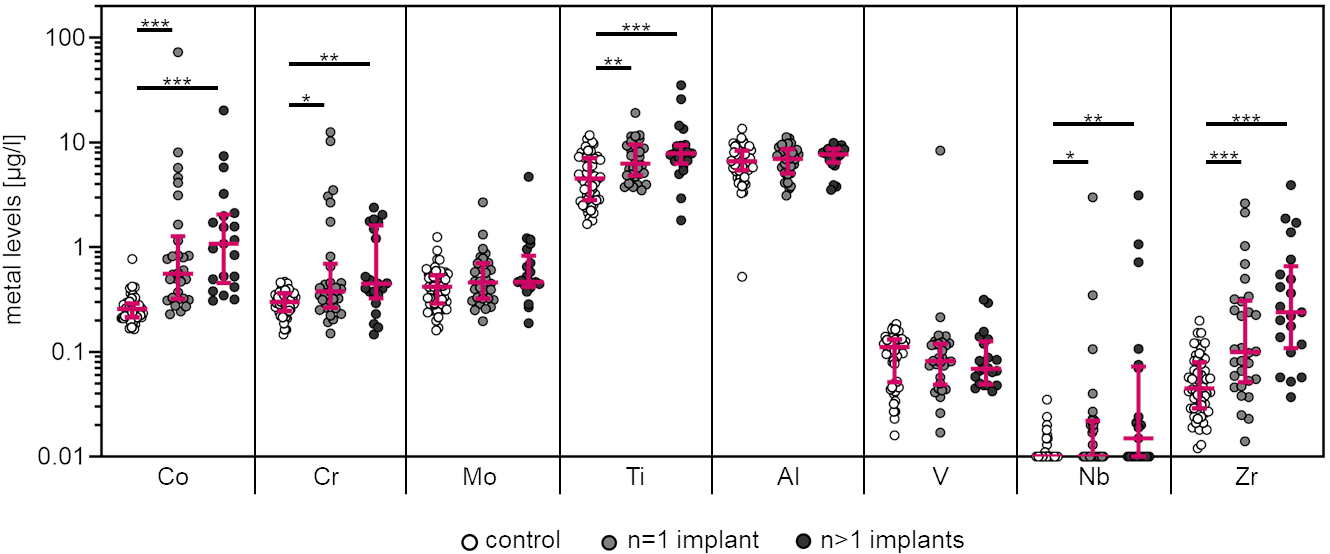


**Figure S2**. Pre-operative multi-metal quantification in whole blood of arthroplasty-naïve patients (control, n = 53), and of patients undergoing revision TKA, who either have no other arthroplasty implant in situ than the index TKA (n=1 implant, n = 30) or who have an/several additional arthroplasty implant/s in situ (n>1 implants, n = 21) [Kruskal-Wallis test with wDunn's multiple comparison test]: *p < 0.05, **p < 0.01, ***p < 0.001].
